# Phytochemical investigation of the *n*-hexane-extracted oil from four umbelliferous vegetables using GC/MS analysis in the context of antibacterial activity

**DOI:** 10.1038/s41598-024-60631-4

**Published:** 2024-05-08

**Authors:** Mostafa H. Baky, Eman M. El-Taher, Dina M. Y. El Naggar, Mostafa B. Abouelela

**Affiliations:** 1 Pharmacognosy Department, Faculty of Pharmacy, Egyptian Russian University, Badr City, 11829 Cairo Egypt; 2 Pharmacognosy Department, Faculty of Pharmacy for Girls, Al Azhar University, Cairo, Egypt

**Keywords:** Apiaceae, Volatiles, GC–MS, Aromatic hydrocarbons, Antibacterial agent, Antimicrobials, Analytical chemistry, Metabolomics

## Abstract

Umbelliferous (Apiaceae) vegetables are widely consumed worldwide for their nutritive and health benefits. The main goal of the current study is to explore the compositional heterogeneity in four dried umbelliferous vegetables viz, celery, coriander, dill, and parsley targeting their volatile profile using gas chromatography-mass spectrometry (GC–MS). A total of 133 volatile metabolites were detected belonging to 12 classes. Aromatic hydrocarbons were detected as the major components of the analyzed vegetables accounting ca. 64.0, 62.4, 59.5, and 47.8% in parsley, dill, celery, and coriander, respectively. Aliphatic hydrocarbons were detected at ca. 6.39, 8.21, 6.16, and 6.79% in parsley, dill, celery, and coriander, respectively. Polyunsaturated fatty acids (PUFA) of various health benefits were detected in parsley and represented by roughanic acid and α-linolenic acid at 4.99 and 0.47%, respectively. Myristicin and frambinone were detected only in parsley at 0.45 and 0.56%. Investigation of antibacterial activity of umbelliferous vegetables *n*-hexane extract revealed a moderate antibacterial activity against Gram-positive and Gram-negative bacteria with higher activity for celery and dill against *Staphylococcus aureus* with inhibition zone 20.3 mm compared to 24.3 mm of the standard antibacterial drug.

## Introduction

Culinary products are herbs and spices used either fresh or dry to improve food aroma and add to the nutritional and health-promoting value of both food and drinks^1,2^. Owing to their richness in essential oils, herbal spices have been recognized as main food ingredients to convey the characteristic flavor and health benefits of different food products and to improve their sensory attributes^3^. Essential oils have been used extensively in traditional medicine owing to their antimicrobial^4^ and immunomodulatory properties^5^. Essential oils are considered raw materials for the pharmaceutical and cosmetic industries owing to their strong preservative and cleansing action^3^. The essential oil composition in herbal spices is directly influenced by several factors including agricultural practices, harvest period, drying, and storage, which affect their food value and or health effects^3^. Seasonal variation is a key factor affecting the level of the bioactive metabolites produced in medicinal plants and, hence, affects their biological properties^6^. Several previous studies have demonstrated that the harvest season can affect essential oil composition by altering both the quantity and quality of the extracted oil^6^.

Umbelliferae (Apiaceae), is a widely distributed plant family with more than 300 genera and about 3000 species native to Central Asia and Europe^1^. Plants of this family are predominantly used globally as aromatic medicinal plants, culinary use, and food additives owing to richness of essential oil as well as a myriad of sensory metabolites and phytochemicals^7^. Among Apiaceae plants, *Apium graveolens* L. (Celery), *Coriandrum sativum* L. (Coriander), *Anethum graveolens* L*.* (Dill), and *Petroselinum crispum* L. (Parsley) are well-known aromatic spices used either fresh or dry^8^. They all possess a high nutritional value, with reduced caloric intake, their leaves, and fresh petioles are mainly consumed in salads as a rich source of minerals and vitamins as A, B, and C^9–11^. They are considered as an excellent source of essential oil with myriad biological activities^12^. Both coriander and dill are used to alleviate gastric complaints^8^. Moreover, parsley and celery were well known to decrease the incidence of diabetic complications^7^. In addition, parsley is beneficial in cardiac and urinary diseases^13^, and celery is used in the management of in atherosclerosis and many infectious diseases^8^.

Several techniques have been employed for the extraction of volatiles from Apiaceae plants for GC–MS analysis including hydro-distillation, steam distillation, solvent extraction, supercritical fluid extraction, solid-phase microextraction, and solvent-free microwave extraction^14,15^. Essential oils as the major secondary metabolites in Apiaceae are of diverse chemical nature including mono-, di-, and sesqui- terpenes, phenylpropanoids, aliphatic aldehydes, aliphatic and aromatic hydrocarbons, octyl esters, and trimethyl-benzaldehydes^16^. They have shown broad biological activities such as antibacterial and antiviral effects, cytotoxic, antifungal, anti-inflammatory, and antioxidant properties^17^. They are also commonly employed as food preservatives to delay the growth of molds and bacteria^18^.

Owing to the increase in consumer demand for functional foods with both nutritional and health value, there is an increase in quality assessment tools to verify both the nutritional and sensory properties of green vegetables^19^. Owing to the high consumption of umbelliferous vegetables and their high nutritional and health values, assessing their metabolites is essential to ensure their quality. Recently, metabolomics tools are widely used to assure the quality of foodstuffs such as fruits and vegetables^20^. Profiling of the volatile metabolites of food products is important for assuring sensory attributes^20^. Gas chromatography coupled with mass spectrometry (GC–MS) analysis is a well-established analytical technique adopted for profiling volatile components in dietary sources^21,22^. Several methods are used for the extraction of volatile metabolites from plant materials including steam distillation, headspace solid phase microextraction (HS-SPME), supercritical fluid extraction, and volatile solvent extractions^23,24^. Compared to thermal-dependent distillation techniques, volatile solvent is applied to extract volatile compounds from plant samples without decomposition and increase the extract yield^23^.

Despite the increase in consumption of Apiaceae green vegetables, there is a lack of recent comparative studies to assess and assure their quality. It only focused on the volatile metabolite of dry fruits^1^. This study aims to assess heterogeneity in volatile compounds in four umbelliferous dried leaves cultivated in Egypt using volatile solvent extraction for the first time coupled with GC–MS analysis, it also aimed to investigate the antimicrobial properties of their essential oils as a source of novel natural antimicrobial agent. Vegetables examined include *Apium graveolens* L. (celery), *Coriandrum sativum* L. (coriander), *Anethum graveolens* L*.* (dill) and *Petroselinum crispum* L. (parsley) to evaluate the variation in their volatile chemical metabolites. To the best of our knowledge, this study provides a comparative study between edible umbelliferous vegetables volatile metabolites for the first time suggesting their uses as functional foods.

## Results and discussion

### Volatile profiling of umbelliferous green vegetables using GC–MS

**Figure 1:**
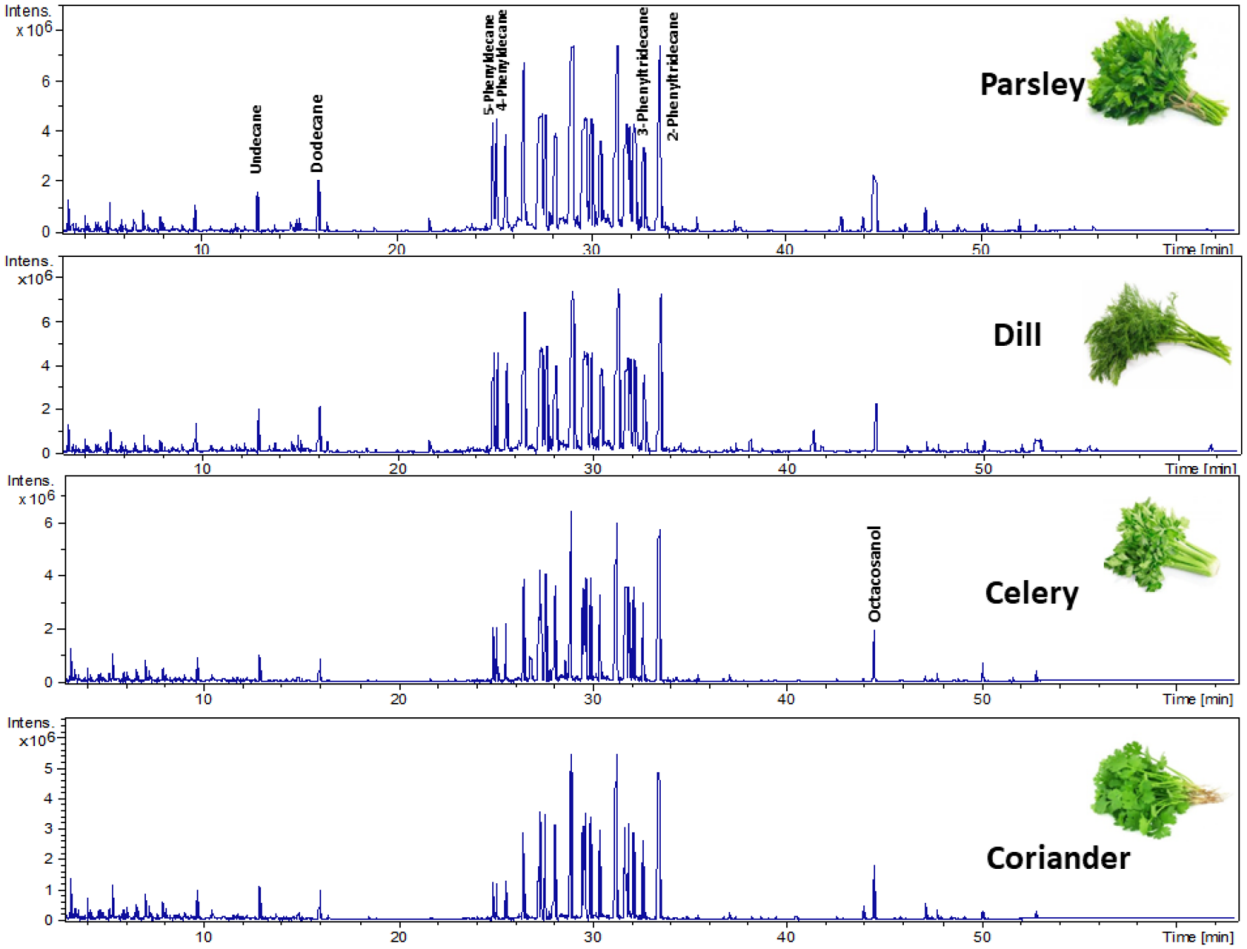
Representative GC–MS chromatograms of volatiles identified in four umbelliferous vegetables.

**Figure 2:**
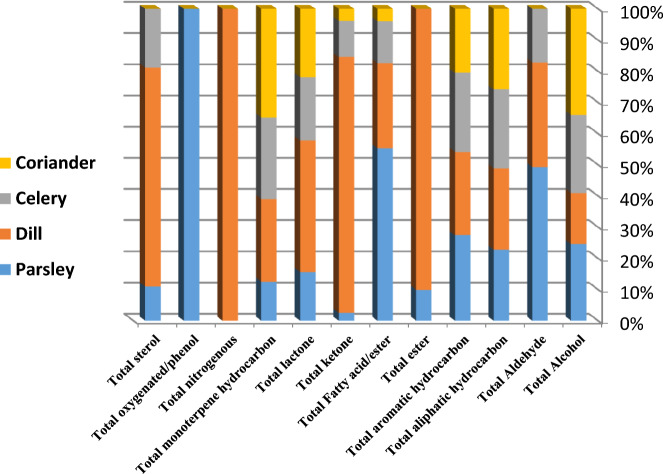
Relative distribution of identified volatile classes in four umbelliferous vegetables.

GC–MS profiling of four umbelliferous green vegetables such as celery, parsley, dill, and coriander revealed the identification of 133 volatile chemical metabolites (Table 1, Fig. 1). The identified volatile metabolites were belonged to 12 classes, viz*.*, alcohols, aldehydes, ketones, aliphatic, alicyclic, and aromatic hydrocarbon, fatty acids/esters, monoterpenes, lactones, oxygenated/phenols, and sterols. GC chromatograms of volatiles identified in four umbelliferous vegetables with some labeled peaks were illustrated in Fig. 1. Each metabolites class includes several compounds with different percentages among the four vegetables, as illustrated in Fig. 2.

**Table 1:** Relative area percentage (%) of volatile metabolites in four dried umbelliferous vegetables analyzed via GC–MS (n = 3).

Peak No	Average Rt (min)	Average RI	Compound name	Class	Parsley (%)	Dill (%)	Celery (%)	Coriander (%)
1	4.821	2069	2-Hexyl-1-octanol	Alcohol	–	0.26 ± 0.02	–	–
2	15.671	1277	2-Butyloctanol	Alcohol	0.3 ± 0.02	0.48 ± 0.02	–	–
3	18.537	1277	2-Hydroxycineol	Alcohol	–	0.12 ± 0.01	–	–
4	35.32	2475	Behenic alcohol	Alcohol	0.11 ± 0.02	–	–	–
5	37.394	2104	Phytol	Alcohol	0.25 ± 0.01	0.19 ± 0.01	–	0.07 ± 0.01
6	43.973	3016	1-Heptacosanol	Alcohol	0.94 ± 0.02	0.19 ± 0.01	0.39 ± 0.01	2.06 ± 0.04
7	46.158	2276	Eicosanol	Alcohol	–	0.27 ± 0.03	–	–
8	47.17	3016	Heptacosanol	Alcohol	1.82 ± 0.05	0.64 ± 0.06	0.72 ± 0.02	2.18 ± 0.01
9	47.355	3118	Octacosanol	Alcohol	–	–	0.1 ± 0.02	0.3 ± 0.02
10	50.065	3016	Heptacosan-1-ol	Alcohol	0.48 ± 0.01	0.93 ± 0.02	2.77 ± 0.02	1.28 ± 0.03
11	52.814	3016	1-Heptacosanol	Alcohol	0.76 ± 0.02	–	0.86 ± 0.02	0.7 ± 0.01
12	60.401	3942	1-Heptatriacotanol	Alcohol	0.1 ± 0.01	0.1 ± 0.04	–	–
Total Alcohol					4.76	3.18	4.84	6.59
13	51.627	1242.5	Hydroxycitronellal	Aldehyde	–	–	0.24 ± 0.08	–
14	51.975	3014	Octacosanal	Aldehyde	0.35 ± 0.01	0.47 ± 0.06	–	–
15	54.77	2221	Eicosanal	Aldehyde	0.34 ± 0.02	–	–	–
Total Aldehyde					0.69	0.47	0.24	0
16	5.735	860.2	Isononane	Aliphatic hydrocarbon	–	–	0.11 ± 0.01	0.18 ± 0.01
17	5.914	1158	6-methyl-2-undecene	Aliphatic hydrocarbon	–	0.31 ± 0.02	–	–
18	5.916	1380	Tridecane, 7-methylene-	Aliphatic hydrocarbon	0.16 ± 0.02	–	–	–
19	6.701	1024	2,5-Dimethylnonane	Aliphatic hydrocarbon	0.32 ± 0.01	–	0.66 ± 0.01	–
20	7.991	799	2,4,4-Trimethyl-1-hexene	Aliphatic hydrocarbon	0.62 ± 0.02	–	1.32 ± 0.01	1.96 ± 0.06
21	8.595	963.8	4-Methylnonane	Aliphatic hydrocarbon	0.05 ± 0.01	–	–	–
22	8.868	1246	3,4-Dimethyl-1-decene	Aliphatic hydrocarbon	–	–	0.32 ± 0.01	0.68 ± 0.01
23	9.792	958.7	2,4,6-Trimethyloctane	Aliphatic hydrocarbon	0.75 ± 0.02	0.65 ± 0.02	0.95 ± 0.05	–
24	10.55	1185	4-Methyl-5-propylnonane	Aliphatic hydrocarbon	–	–	0.34 ± 0.01	–
25	10.553	1259	4,6-Dimethyldodecane	Aliphatic hydrocarbon	0.17 ± 0.01	–	–	0.48 ± 0.05
26	10.565	1055	2,3-Dimethylnonane	Aliphatic hydrocarbon	–	0.41 ± 0.05	–	–
27	11.641	1027	2,5-Dimethylnonane	Aliphatic hydrocarbon	0.28 ± 0.01	–	–	–
28	11.645	860	4-Ethylheptane	Aliphatic hydrocarbon	–	0.32 ± 0.02	–	–
29	11.844	1065.9	2-Methyldecane	Aliphatic hydrocarbon	–	–	0.18 ± 0.01	0.27 ± 0.02
30	11.867	1129	3,6-Dimethyldecane	Aliphatic hydrocarbon	–	0.47 ± 0.02	–	–
31	12.045	1072.4	3-Methyldecane	Aliphatic hydrocarbon	0.09 ± 0.01	–	0.11 ± 0.01	0.17 ± 0.01
32	12.475	1393	1,13-Tetradecadiene	Aliphatic hydrocarbon	–	0.43 ± 0.02	–	–
33	12.665	1040	1-Methyl-3-propylcyclohexane	Aliphatic hydrocarbon	–	–	0.14 ± 0.01	–
34	12.97	955	2,4,6-Trimethyloctane	Aliphatic hydrocarbon	–	–	0.97 ± 0.02	–
35	12.995	1100	Undecane	Aliphatic hydrocarbon	0.75 ± 0.01	–	–	1.46 ± 0.02
36	13.008	1027	2,5-Dimethylnonane	Aliphatic hydrocarbon	–	1.26 ± 0.02	–	–
37	13.471	1162	2-methyl-Decalin	Aliphatic hydrocarbon	0.22 ± 0.01	–	0.18 ± 0.04	0.3 ± 0.02
38	13.949	1159	2-methyldecalin	Aliphatic hydrocarbon	–	0.63 ± 0.05	–	–
39	14.721	1156	Methylundecane	Aliphatic hydrocarbon	0.6 ± 0.01	0.91 ± 0.1	0.08 ± 0.02	–
40	14.992	1170	2-Methylundecane	Aliphatic hydrocarbon	0.51 ± 0.1	0.99 ± 0.05	–	–
41	16.108	1200	Dodecane	Aliphatic hydrocarbon	1.36 ± 0.02	1.54 ± 0.05	0.8 ± 0.02	1.29 ± 0.01
42	21.761	1400	Tetradecane	Aliphatic hydrocarbon	0.26 ± 0.01	0.29 ± 0.03	–	–
43	50.321	4000	Tetracontane	Aliphatic hydrocarbon	0.18 ± 0.02	–	–	–
44	52.995	4395	Tetratetracontane	Aliphatic hydrocarbon	0.07 ± 0.01	–	–	–
Total aliphatic hydrocarbon					6.39	8.21	6.16	6.79
45	3.678	782	1,3-Dimethylcyclohexane	Alicyclic hydrocarbon	0.18 ± 0.02	0.26 ± 0.04	0.29 ± 0.01	0.41 ± 0.01
46	4.814	847	1,2,4-trimethylcyclohexane	Alicyclic hydrocarbon	0.29 ± 0.01	–	0.39 ± 0.02	–
47	5.995	853	1,2,4-trimethylcyclohexane,	Alicyclic hydrocarbon	0.19 ± 0.01	–	0.39 ± 0.02	–
48	6.22	883	1-Ethyl-4-methylcyclohexane	Alicyclic hydrocarbon	0.17 ± 0.05	0.16 ± 0.01	0.39 ± 0.02	0.58 ± 0.05
49	7.435	930.5	Propylcyclohexane	Alicyclic hydrocarbon	–	–	0.35 ± 0.01	–
50	7.445	1114	2-Propyl-1,1-dimethylcyclohexane	Alicyclic hydrocarbon	0.15 ± 0.04	–	–	–
51	7.77	1682	1,3-Dimethyl-(3,7-dimethyloctyl)cyclohexane	Alicyclic hydrocarbon	0.09 ± 0.02	0.09 ± 0.01	0.21 ± 0.02	0.31 ± 0.01
52	8.359	837.8	Cyclogeraniolane	Alicyclic hydrocarbon		–	0.23 ± 0.02	0.35 ± 0.22
53	10.749	1318	1-Pentyl-2-propylcyclopentane	Alicyclic hydrocarbon	–	–	0.14 ± 0.02	
54	10.77	1119	1,2-Dipropylcyclopentane	Alicyclic hydrocarbon	0.07 ± 0.01	–	–	0.35 ± 0.08
55	10.885	1361	1,2,4,5-Tetraethylcyclohexane	Alicyclic hydrocarbon	–	0.13 ± 0.03	–	–
56	12.317	907.1	1-Methyl-1-ethylcyclohexane	Alicyclic hydrocarbon	0.17 ± 0.04	–	–	–
57	12.471	1124	4-Pentenylcyclohexane	Alicyclic hydrocarbon	–	–	0.15 ± 0.01	–
58	12.595	1114	(1-Ethylpropyl)cyclohexane	Alicyclic hydrocarbon	–	0.05 ± 0.01	–	–
59	12.675	1008.9	Isobutylcyclohexane	Alicyclic hydrocarbon	0.11 ± 0.01	–	–	–
60	12.688	1121	Amylcyclohexane	Alicyclic hydrocarbon	–	0.19 ± 0.69	–	–
61	13.865	1760	Undecylcyclohexane	Alicyclic hydrocarbon	0.2 ± 0.03	–	–	0.27 ± 0.85
62	13.879	1121	Amylcyclohexane	Alicyclic hydrocarbon	–	0.32 ± 0.0.3	0.17 ± 0.02	–
Total alicyclic hydrocarbon					1.62	1.2	2.71	2.27
63	5.215	854	Ethylbenzene	Aromatic hydrocarbon	0.17 ± 0.98	0.06 ± 0.55	0.38 ± 0.06	0.55 ± 0.05
64	9.055	962	Mesitylene	Aromatic hydrocarbon	–	–	0.14 ± 0.03	0.18 ± 0.04
65	23.701	1814	6-phenyltridecane	Aromatic hydrocarbon	–	0.08 ± 0.04	–	–
66	24.196	2618	4-phenyleicosane	Aromatic hydrocarbon	0.06 ± 0.01	0.11 ± 0.51	1.22 ± 0.44	0.86 ± 0.50
67	25.014	1526	5-Phenyldecane	Aromatic hydrocarbon	2.44 ± 0.51	1.74 ± 0.21	1.26 ± 0.22	0.86 ± 0.09
68	25.24	1534	4-phenyldecane,	Aromatic hydrocarbon	2.13 ± 0.66	1.56 ± 0.55	1.22 ± 0.50	0.86 ± 0.61
69	25.695	1553	3-Phenyldecane	Aromatic hydrocarbon	3.02 ± 0.81	2.24 ± 0.36	1.69 ± 0.53	1.28 ± 0.81
70	26.255	1814	6-phenyltridecane	Aromatic hydrocarbon	–	–	0.28 ± 0.07	–
71	26.59	1588	2-phenyldecane	Aromatic hydrocarbon	4.86 ± 1.08	3.69 ± 0.78	2.5 ± 0.44	2.02 ± 0.02
72	26.98	1504	2-Phenyl-2-butene	Aromatic hydrocarbon	0.65 ± 0.08	–	–	–
73	27.513	1626	5-phenylundecane	Aromatic hydrocarbon	6.24 ± 0.89	5.2 ± 0.45	6 ± 0.71	5.2 ± 0.80
74	27.758	1636	4-phenylundecane	Aromatic hydrocarbon	3.54 ± 0.78	2.9 ± 0.90	3.78 ± 0.17	3.37 ± 0.01
75	27.879	1791	2-Phenyldodecane	Aromatic hydrocarbon	–	–	0.31 ± 0.08	–
76	28.258	1659	3-Phenylundecane	Aromatic hydrocarbon	5.13 ± 0.25	4.22 ± 0.05	4.54 ± 0.06	0.25 ± 0.08
77	28.959	1692	2-phenylundecane	Aromatic hydrocarbon	6.32 ± 0.21	5.35 ± 0.95	6.24 ± 0.80	5.74 ± 0.88
78	29.708	1722	5-Phenyldodecane	Aromatic hydrocarbon	–	–	7.38 ± 0.88	6.85 ± 0.50
79	29.749	1719	6-Phenyldodecane	Aromatic hydrocarbon	6.75 ± 0.47	5.83 ± 0.22	0.37 ± 0.05	0.18 ± 0.06
80	30.086	1735	4-Phenyldodecane	Aromatic hydrocarbon	4.09 ± 0.06	6.04 ± 1.51	4.28 ± 0.88	3.97 ± 0.98
81	30.301	1462	2-phenyl-3-propyl-Hexane	Aromatic hydrocarbon	0.82 ± 0.01	–	–	–
82	30.573	1755	3-Phenyldodecane	Aromatic hydrocarbon	4.24 ± 0.09	3.95 ± 0.12	4.42 ± 0.52	4.07 ± 0.58
83	30.758	1504	2-Phenyl-2-butene	Aromatic hydrocarbon	–	–	0.46 ± 0.09	–
84	30.811	1791	2-Phenyldodecane	Aromatic hydrocarbon	1.16 ± 0.08	4.85 ± 1.61	0.41 ± 0.06	0.34 ± 0.88
85	31.771	1814	6-phenyltridecane	Aromatic hydrocarbon	–	–	4.68 ± 0.28	4.35 ± 1.58
86	31.87	1818	7-Phenyltridecane	Aromatic hydrocarbon	4.58 ± 0.05	0.89 ± 0.01	0.46 ± 0.04	0.46 ± 0.05
87	32.033	1821	5-phenyltridecane	Aromatic hydrocarbon	2.82 ± 0.50	6.19 ± 1.98	3.61 ± 0.79	3.4 ± 0.98
88	32.286	1833	4-phenyltridecane	Aromatic hydrocarbon	3.05 ± 0.65	2.6 ± 0.05	3.12 ± 0.88	3.21 ± 1.85
89	32.783	1866	3-phenyltridecane	Aromatic hydrocarbon	3.97 ± 0.52	3.41 ± 0.06	4.04 ± 0.55	6.8 ± 1.05
90	33.552	1894	2-Phenyltridecane	Aromatic hydrocarbon	–	3.77 ± 0.055	4.76 ± 0.45	0.09 ± 0.02
91	33.775	1922	7-phenyltetradecane	Aromatic hydrocarbon	–	0.06 ± 0.05	–	–
92	34.745	1966.9	3-phenyltetradecane	Aromatic hydrocarbon	0.16 ± 0.01	–	–	–
Total aromatic hydrocarbon					64	62.45	59.19	47.83
93	4.075	804.9	Butyl acetate	Ester	–	0.3 ± 0.08	–	–
94	11.365	2540	Diethylhexyl carbonate	Ester	–	0.07 ± 0.02	–	–
95	35.15	2080	Malonic acid, isobutyl undecyl ester	Ester	–	0.12 ± 0.01	–	–
96	50.572	2574	Behenyl acetate	Ester	–	0.24 ± 0.04	–	–
97	56.045	2579	Carbonic acid, eicosyl prop-1-en-2-yl ester	Ester	0.08 ± 0.01	–	–	–
98	58.13	3058.3	Arachidyl benzoate	Ester	0.04 ± 0.02	0.06 ± 0.01	–	–
Total ester					0.12	0.79	–	–
99	33.685	1908	Methyl palmitate	Fatty acid/Ester	–	0.28 ± 0.54	–	–
100	33.764	1989	Roughanic acid	Fatty acid/Ester	4.99 ± 0.05	–	–	–
101	34.399	1942	Palmitic acid	Fatty acid/Ester	0.47 ± 0.02	1.58 ± 0.02	1.26 ± 0.03	0.42 ± 0.01
102	37.646	2095	Linoleic acid	Fatty acid/Ester	–	0.54 ± 0.07	–	–
103	37.669	2102	α-Linolenic acid	Fatty acid/Ester	0.47 ± 0.09	–	–	–
104	38.726	2157	Butyl palmitate	Fatty acid/Ester	–	0.23 ± 0.01	–	–
105	40.635	2618	4-Hydroxybutyl stearate	Fatty acid/Ester	–	0.19 ± 0.08	0.18 ± 0.01	–
106	43.471	2166	Ethyl linolenate	Fatty acid/Ester	–	0.12 ± 0.01	–	–
Total Fatty acid/ester					5.93	2.94	1.44	0.42
107	8.003	1031	1-(2,2-dimethylcyclopentyl)ethanone,	Ketone	–	0.6 ± 0.01	–	–
108	9.112	1089	1-(1-Methyl-cyclohexyl)-ethanone	Ketone	0.24 ± 0.09	–	–	–
109	26.891		Isobenzofuranone	Ketone	–	–	1 ± 0.04	–
110	51.277	3089.9	Nonacosan-10-one	Ketone	–	0.1 ± 0.02	–	0.27 ± 0.01
111	52.711	3090	Ginnone	Ketone	–	5.24 ± 0.44	–	–
112	53.015	3040	2-Nonacosanone	Ketone	–	–	0.1 ± 0.02	0.09 ± 0.01
113	55.441	2045	10-Nonadecanone	Ketone	–	1.83 ± 0.01	–	–
Total ketone					0.24	7.77	1.1	0.36
114	36.769	1920	γ -Tetradecalactone	Lactones	–	–	0.13 ± 0.05	0.14 ± 0.01
115	36.789	2106	γ-Palmitolactone	Lactones	–	0.17 ± 0.05	–	–
116	37.105	1521	Persicol	Lactones	–	0.1 ± 0.03	–	–
117	47.391	2178	γ-Stearolactone	Lactones	0.1 ± 0.05	–	–	–
Total lactone					0.1	0.27	0.13	0.14
118	7.349	989	1-Methyl-2-propylcyclohexane	Monoterpene hydrocarbon	0.1 ± 0.01	0.06 ± 0.05	0.21 ± 0.05	0.31 ± 0.05
119	8.414	967.5	1-Ethyl-2,3-dimethylcyclohexane	Monoterpene hydrocarbon	0.1 ± 0.01	0.1 ± 0.02	–	–
120	8.779	981.6	*p*-Menthane	Monoterpene hydrocarbon	–	0.32 ± 0.02	–	–
121	9.115	973	*p*-Menthane isomer	Monoterpene hydrocarbon	–	0.29 ± 0.02	–	0.59 ± 0.03
122	9.498	1040	1-Methyl-3-propylcyclohexane	Monoterpene hydrocarbon	–	–	0.19 ± 0.01	–
123	10.346	983	1-Methyl-2-propylcyclohexane	Monoterpene hydrocarbon	0.08 ± 0.01	0.15 ± 0.04	0.18 ± 0.02	0.22 ± 0.01
Total monoterpene hydrocarbon					0.28	0.92	0.58	1.12
124	38.145	2021	Palmitamide	Nitrogenous	–	0.37 ± 0.05	–	–
125	41.352	2375	Oleamide	Nitrogenous	–	1.44 ± 0.01	–	–
126	41.786	2349	Stearamide	Nitrogenous	–	0.14 ± 0.01	–	–
Total nitrogenous					–	1.95	–	–
127	23.943	1516	Myristicin	Oxygenated/phenolic	0.45 ± 0.04	–	–	–
128	42.864	1498	Frambinone	Oxygenated/phenolic	0.56 ± 0.05	–	–	–
Total oxygenated/phenol					1.01	–	–	–
129	52.443	3138	Stigmasterol	Sterol	–	–	0.06 ± 0.01	–
130	54.944	3142	β-Stigmasterol	Sterol	–	0.69 ± 0.01	0.22 ± 0.02	–
131	55.882	3173	γ-Sitosterol	Sterol	–	0.68 ± 0.43	0.20 ± 0.03	–
132	56.73	3345	Stigmast-7-en-3-ol	Sterol	–	0.18 ± 0.02	–	–
133	57.047	3090	Betulinal	Sterol	0.28 ± 0.02	0.24 ± 0.05	–	–
	Total sterol				0.28	1.79	0.48	–

#### Aromatic hydrocarbons

Aromatic hydrocarbons were identified as the major volatile class detected in the four umbelliferous vegetables and accounted for 64, 62.45, 59.19, and 47.83% in parsley, dill, celery, and coriander, respectively (Fig. 2). Phenyl-undecane derivatives, a group of aromatic hydrocarbons with antifungal and antibacterial activities were reported at higher levels in green vegetables^23^. The most abundant aromatic detected in all vegetables was 2-phenylundecane (peak 77) which amounted to 6.3, 5.3, 6.2, and 5.7% in parsley, dill, celery, and coriander, respectively. 5-Phenylundecane (peak 73) was detected at high levels in all umbelliferous vegetables with an average concentration of 5.6% and was found to be more abundant in parsley and celery leaves. 6-Phenyldodecane (peak 79) was detected at a higher level in both parsley and dill at 6.7 and 5.8%, respectively, compared to trace levels in celery and coriander. Controversy, 5-phenyldodecane (peak 78), an antifungal volatile compound^25^ was detected at higher levels only in celery and coriander at ca. 4.6 and 4.3%, respectively. 2-phenyldecane (peak 71) was detected at high levels in parsley and dill at ca. 4.8 and 3.6%, respectively. 3-phenylundecane (peak 76) was detected at level range ca. 4.2–5.1% in parsley, dill, and celery, compared to trace levels in coriander. 2-phenyltridecane (peak 89) was detected at relatively high levels in dill and celery at levels 3.77 and 4.76%, respectively. Aromatic hydrocarbons and their derivatives have been reported in several plants essential oil and revealed good antibacterial activity^26^. Among aromatic hydrocarbons, decane derivatives showed antifungal and antibacterial activities^27^.

#### Aliphatic hydrocarbons

Aliphatic hydrocarbons were identified as the second major group of metabolites identified in the four umbelliferous vegetables represented by 29 metabolites and accounted *ca*. 6.4, 8.2, 6.1, and 6.7% in parsley, dill, celery, and coriander, respectively. Recently, aliphatic hydrocarbons derived from vegetable oils are economicaly important as a source of green biofuel^28^*.* Dodecane (peak 41) was detected levels 1.3, 1.4, 0.8, and 1.3%, in parsley, dill, celery, and coriander, respectively. Dodecane, a liquid alkane hydrocarbon of the paraffin series used mainly in green diesel biofuel production^29^. Undecane (peak 35) was identified only in parsley and coriander at 0.7 and 1.4%, respectively. Undecane is a naturally occurring alkane hydrocarbon with diverse biological activities as anti-inflammatory, antiallergic and immunosuppressant^23^. 2-Methyldecane detected in both celery and coriander was previously reported in *Opuntia ficus indica* volatiles^30^.

#### Alicyclic hydrocarbons

Alicyclic hydrocarbons, also known as cycloalkanes, are saturated hydrocarbons with one or more rings with attached alkyl side chains^31^. Alicyclic hydrocarbons are common naturally occurring compounds mainly found in crude plant oil^31^. Alicyclic hydrocarbons were detected at lower levels in umbelliferous vegetables leaf at level range *ca.* 1.2–2.7%. These compounds have been associated with various therapeutic activities including antibacterial, antiviral, and hepatoprotective^32^. 17 alcyclic hydrocarbons were detected in umbelliferous vegetables among which 1,3-dimethylcyclohexane and 1-ethyl-4-methylcyclohexane were detected in all umbelliferous vegetables at level range 0.1–0.6%. Cyclogeraniolane previously detected in *Horwoodia dicksoniae* aerial parts^33^, was detected in celery and coriander at level 0.23 and 0.35%, respectively.

#### Alcohols

Alcohols were detected at comparable levels in all umbelliferous vegetables by 4.7, 3.1, 4.8, and 6.5 in parsley, dill, celery, and coriander, respectively. Heptacosanol (Peaks 6, 8, and 11) was identified in all umbelliferous vegetables at a concentration range of 1.7–5.5% and was abundant in both celery and coriander at ca. 3.8 and 5.5%, respectively. 1-Heptacosanol is a fatty alcohol well-known for its anti-bacterial properties^34^. Phytol, a diterpene alcohol with a pleasant aroma and myriad biological activities such as antioxidant, anti-inflammatory, and antimicrobial^23^, was only detected in parsley and dill only. Moreover, behenic alcohol, a fatty alcohol widely used in the cosmetics and pharmaceutical industry^35^ were only detected in parsley.

#### Fatty acids/esters

Fatty acids/esters were detected at a relative amount in the volatile blend of the umbelliferous vegetables at the range of 0.4–5.9% and were highly abundant in parsley by 5.9%. Saturated and unsaturated fatty acids and/or esters were detected in umbelliferous vegetables. Roughanic acid (peak 100) and α-linolenic acid (peak 103) are important polyunsaturated fatty acids (PUFA) and were detected only in parsley at concentrations of 4.99 and 0.47%, respectively. Dietary PUFA including ω-3 and ω-6 fatty acids are important for promoting human health owing to their beneficial medicinal and nutritional attributes^36^. PUFA plays a pivotal role as an anti-inflammatory, anti-cancer, anti-aging, and hypolipidemic, they reduce the risk of developing cardiovascular diseases, protect against osteoarthritis and various auto-immune disorders^37^. Palmitic acid (peak 101) was detected in all umbelliferous vegetables and was more abundant in dill and celery at 1.58 and 1.26%, respectively. Fatty acid esters such as butyl palmitate and ethyl linolenate were detected only in dill leaves. Methyl palmitate was detected at trace levels in umbelliferous vegetables and in accordance with other previous reports^38^. Organic acid esters were detected only in parsley and dill at much lower levels at ca. 0.12 and 0.79% represented by butyl acetate, behenyl acetate at 0.3 and 0.2%, respectively.

#### Aldehydes/ketones/lactones

Aldehydes were detected at trace levels in only three vegetables i.e., parsley, dill, and celery at ca. 0.69, 0.47, and 0.24%, respectively. Octacosanal (peak 14) was identi-fied in parsley and dill only, hydroxy citronellal was detected only in celery, while eicosanal was detected only in parsley. Compared to aldehydes, ketones were detected in all umbelliferous vegetables and were highly abundant in dill at 7.77%. Gin-none(nonacosan-10-one) (peak 111) was detected as the major ketone in dill at ca. 5.24%. This result was following the previous study done by Peerakam et al. which identified nonacosan-10-one as the major component with antioxidant capacity in dill^39^. Moreover, 10-nonadecanone (1.83%) was detected in dill only. Lactones were detected at trace levels in all umbelliferous vegetables ranging from 0.1–0.27%. Persicol (peak 116) and γ-palmitolactone (peak 115) were detected only in dill at 0.1%, with γ-stearolactone being only detected in parsley.

#### Nitrogenous compounds/oxygenated phenols

Nitrogenous compounds were detected only in dill at *ca.* 1.95% (Fig. 2) while being absent in other umbelliferous vegetables. Oleamide (peak 125) was detected with a higher concentration in dill at 1.44%. Oleamide is a fatty acid amide of biological significance known for its anti-inflammatory activity^40^. Myristicin (peak 127) and frambinone (peak 128) were detected only in parsley at levels 0.45 and 0.56%, respectively. Myristicin is a natural compound identified in nutmeg, parsley, carrots, and peppers with potential health benefits such as antioxidant, anti-inflammatory, antiproliferative, and antimicrobial activities^13^. In a previous study done by Farouk et al., myristicin was identified as the major constituents in parsley volatiles analyzed by HS-SPME which support its detection in parsley^41^. Frambinone is a phenolic compound detected in *Zingiber officinale* and *Zingiber montanum* and is known for its antibacterial activity^42^.

#### Terpenes/sterols

Monoterpene compounds were detected in all umbelliferous vegetables at low levels ranging from 0.78 to 2.17% and were found to be abundant in coriander. Sterols were detected at trace levels in parsley and celery and relatively higher levels in dill at 1.79% and were absent in coriander. β-Stigmasterol and γ-sitosterol (peaks 130 and 131, respectively) were detected in both dill and celery. Plant sterols are highly abundant in vegetable oils, and nuts and play a pivotal role in human health as potent hypolipidemic candidates^23^. Moreover, phytosterols possess potent anti-inflammatory potential and help in treatment of several ailments such as rheumatoid arthritis, inflammatory bowel diseases, multiple sclerosis, asthma, and cardiovascular diseases^43^.

To the best of our knowledge and compared to the previous studied, it is the first time to profile umbelliferous vegetables volatiles using solvent extraction method. Solvent extraction method with several objectives including availability, lower cost, and being volatile and easily removed from the extract, provides a suitable method for profiling of vegetables oil extract. Such results add to the chemical profiling of these important vegetables used widely in food production.

### PCA analysis of umbelliferous samples' volatile metabolites

**Figure 3:**
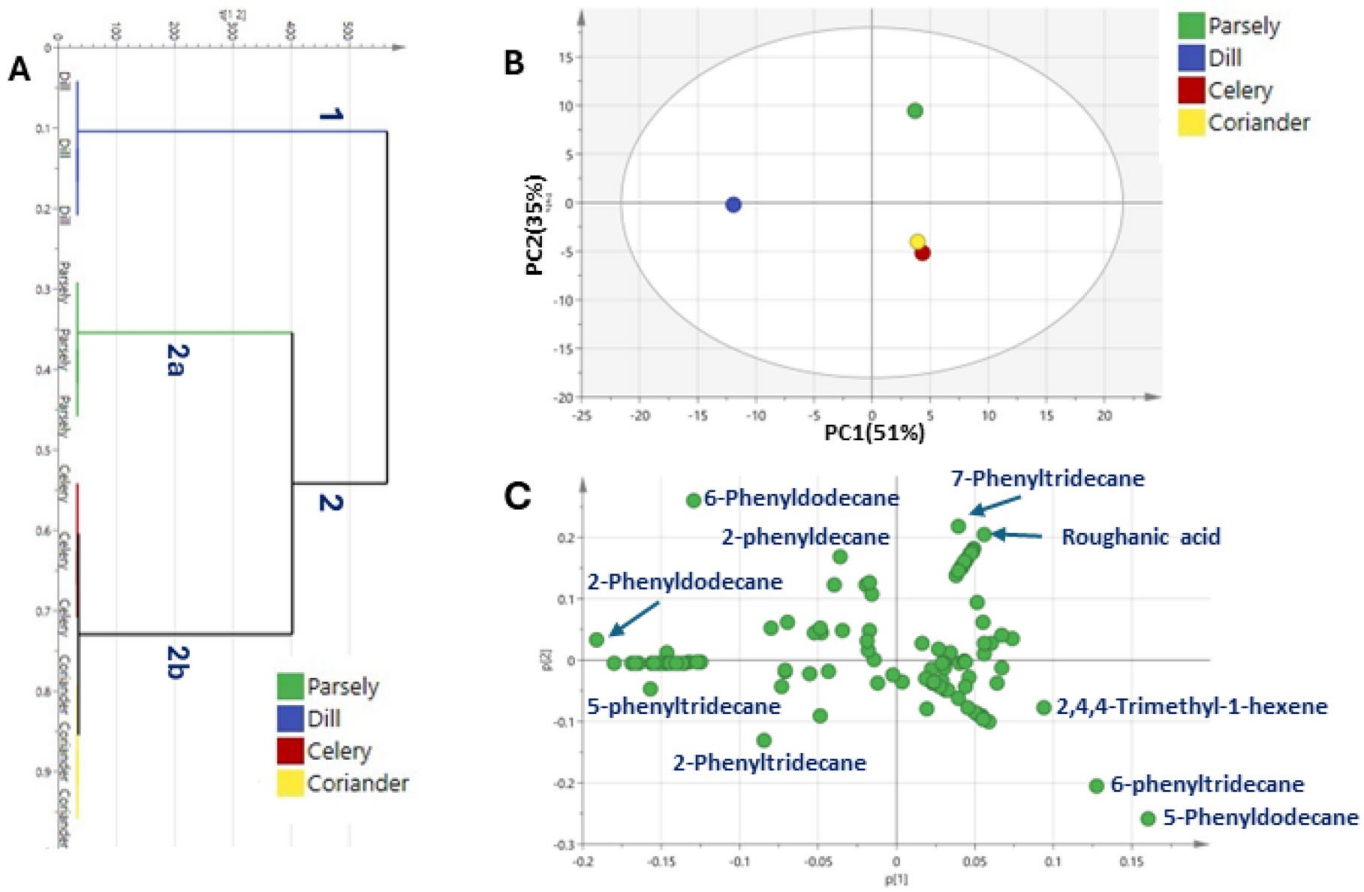
Unsupervised multivariate data analyses of four umbelliferous vegetables volatile compounds detected using GCMS (n = 3). (**A**) HCA plot. (**B**) PCA score plot of PC1 vs. PC2 scores. (**C**) The respective loading plot for PC1 and PC2, provide peak assignments. The metabolite clusters are placed in two-dimensional space at the distinct locations defined by two vectors of principal component PC1 = 51% and PC2 = 35%.

Multivariate data analysis using Hierarchical cluster analysis (HCA) and principal component analysis (PCA) were used for better assessment of metabolite distribution among the four umbelliferous samples (Fig. 3). HCA depicted a dendrogram in which two distinct clusters (Fig. 3A) were observed, with dill clustered in group 1, whereas other samples were clustered in two subdivisions from group 2 where parsley was clustered at subgroup 2a and both celery and coriander were clustered under subgroup 2b. The clustering of three samples together indicated the weakness of the HCA model in the characterization of metabolite heterogeneity. A PCA model (Fig. 3B) showed discrimination of dill clusters at the left side of PC1. In contrast, towards the right side of PC1 showed two clusters: one for parsley on the positive side versus celery and coriander on the negative side of PC2. The corresponding loading plot Fig. 3C revealed that 2-phenyldodecane, 5-phenyltridecane, 2-phenyldecane, 6-phenyldodecane, and 2-phenyltridecane were enriched in dill samples, alongside 7-Phenyltridecane and roughanic acid were more enriched parsley while 6-phenyltridecane, 5-phenyldodecane, and 2,4,4-Trimethyl-1-hexene were enriched in both celery and coriander and accounting for their segregation.

### Antibacterial activity

Recently, the increasing in mutation and resistance of microorganisms against synthetic antimicrobial agent enhanced searching for new and safe antimicrobial agents within herbal plants^44^. Several studies documented the antimicrobial action of essential oils and their constituents^45^. Umbelliferous vegetables are considered as an excellent source of essential oil which possess antimicrobial properties^12^. Comparative investigation of the antibacterial activity of dried umbelliferous vegetables *n*-hexane extracted oil (Table 2) revealed that celery and dill *n*-hexane extracts showed promising anti-microbial activity with a 20.3- and 18.5-mm zone of inhibition, respectively, and as compared to those of parsley and coriander extracts with inhibition zone 12.5 and 11.2 mm, respectively, against *S. aureus*. The minimum inhibitory concentration (MIC) of umbelliferous vegetables compared to the standard antibacterial was listed in Table 3. Moreover, celery *n*-hexane extract exhibited strong inhibition against *S. mutants* with an inhibition zone of 17.4 mm and MIC of 1.85 µg/mL followed by coriander (15.3 mm and MIC 1.98 µg/mL), compared to parsley, and dill (MIC 3.9 µg/mL) extracts, their inhibition zones were 15.3, 11.5, and 12.9 mm, respectively. Among all extracts, celery showed the highest activity with an inhibition zone of 18.3 mm and MIC 0.98 µg/mL against *B. subtilis* compared to inhibition zone of 12.4 and 12.5 mm for coriander and dill (MIC 1.98 µg/mL), respectively. Moreover, coriander *n*-hexane extract only showed activity against *B. cereus* with a zone of inhibition of 9.5 mm. While dill and celery *n*-hexane extract only showed activity against *E. faecalis* with inhibition zone of 17.3 and 15.1 mm, and MIC 10.7 and 7.81 µg/mL, respectively. Celery *n*-hexane extract showed activity against gram-negative bacteria *E. cloacae* with an inhibition zone of 17.2 mm and MIC of 7.81 µg/mL, meanwhile, dill, parsley, and coriander showed 10-, 10.5-, and 9.5-mm zone of inhibition, and MIC of 13.95, 15.63, and 15.63 µg/mL, respectively. The extracts showed no activity against *E. coli*, and *S. typhimurium*. The essential oils obtained from the four vegetables under investigation have been reported to inhibit a broad spectrum of microorganisms^46,47^. Such activity may be attributed to its richness with aromatic hydrocarbons specially decane derivatives which reported for its antibacterial activity^27^. Essential oils from dill and coriander have been reported to inhibit a broad spectrum of microorganisms as the crude essential oils from these plant species were effective against several bacteria^46,47^. Dodecane derivatives which were detected in umbelliferous vegetables were reported for antibacterial activity^48^. In agreement with previous reports, strength and spectrum of activity varied between plant species and gram-positive bacteria were generally more sensitive to the effects of the oils. A previous study done by Mansureh Ghavam, 2022 revealed the antibacterial activity of the essential oil of *Rosmarinus officinalis*^49^ and *Hymenocrater incanus* and *Dracocephalum kotschyi*^50^. Moreover, the essential oils of celery and coriander were reported for their antimicrobial activity showing coriander more effective than celery^46,47^. They also had shown antibacterial activities against different foodborne pathogens^51^. Phytol detected in umbelliferous vegetable was reported to be effective against Gram-positive bacteria^52^. The results of this study were found to be in agreement with the previous reports, where the spectrum of activity varied between those species, with gram-positive bacteria generally were more sensitive to the essential oil effect than the gram-negative ones^46^. Parsley, and dill exhibited antibacterial activity when added to the Kareish cheese with improvement of consumer acceptance^53^. The study confirmed that the aforementioned vegetables exerted antimicrobial effect against infectious microorganisms in humans and on those that can cause food spoilage^53^.

**Table 2:** Comparative antibacterial activity of four umbelliferous green vegetables *n*-hexane extract expressed as zone of inhibition (mm) (n = 3). Statistical analysis was carried out by one-way ANOVA with p ≤ 0.05 indicates statistical significance.

Evaluated microorganism/sample	Parsley	Coriander	Dill	Celery	Standard
Gram positive bacteria					
*Staphylococcus aureus* ATCC 25923	12.5 ± 0.2**	11.2 ± 0.5^ ns^	18.5 ± 0.3***	20.3 ± 0.3***	24.3 ± 0.5
*Bacillus subtilis* RCMB 015 (1) NRRL B-543	NA	12.4 ± 0.4*	12.5 ± 0.4*	18.3 ± 0.5***	26.3 ± 0.5
*Bacillus cereus* RCMB 027 (1)	NA	9.5 ± 0.3^ ns^	NA	NA	24.8 ± 0.6
*Streptococcus mutans* RCMB 017 (1) ATCC 25175	11.5 ± 0.3*	15.3 ± 0.4***	12.9 ± 0.4*	17.4 ± 0.4***	22.2 ± 0.4
*Enterococcus faecalis* ATCC 29212	NA	NA	17.3 ± 0.4*	15.1 ± 0.4*	25.8 ± 0.4
Gram negative bacteria					
*Enterobacter cloacae* RCMB 001(1) ATCC 23355	10.5 ± 0.4*	9.5 ± 0.3^ ns^	10 ± 0.5^ ns^	17.2 ± 0.6*	29.8 ± 0.7
*Escherichia coli* ATCC 25922	NA	NA	NA	NA	30.5 ± 0.4
*Salmonella typhimurium* RCMB 006	NA	NA	NA	NA	17.3 ± 0.5

*NA* no activity.

^ns^p > 0.05, *p ≤ 0.05, **p $$\le$$ 0.01, ***p ≤ 0.001.

**Table 3:** Minimum Inhibitory Concentration (MIC) for dill and celery calculated as µg/mL.

Evaluated micro-organism/sample	Parsley	Dill	Celery	Coriander	Standard
Gram-positive bacteria					
*Staphylococcus aureus* ATCC 25923	3.9	1.85	1.41	3.9	0.98
*Bacillus subtilis* RCMB 015 (1) NRRL B-543	NA	1.98	0.98	1.98	0.49
*Streptococcus mutants* RCMB 017 (1) ATCC 25175	3.9	3.9	1.85	1.98	0.98
*Enterococcus faecalis* ATCC 29212	NA	10.7	7.81	NA	3.9
Gram-negative bacteria					
* Enterobacter cloacae* RCMB 001(1) ATCC 23355	15.63	13.95	7.81	15.63	3.9

## Conclusion

The volatile composition heterogeneity in the *n*-hexane extracted oil from dried leaves of parsley, dill, celery, and coriander was introduced herein. A total of 133 volatile metabolites were identified by using the GC–MS profiling technique. Aromatic hydrocarbons were identified as the major volatile class detected in the four umbelliferous vegetables. Aliphatic hydrocarbons were identified as the second abundant group of metabolites in the four umbelliferous vegetables. The results manifested both qualitative and quantitative differences between the four studied vegetables. Moreover, the *n*-hexane extract of the four vegetables showed antibacterial activity against Gram-positive and Gram-negative selected strains. Celery and dill were revealed to be the most effective against tested bacteria. To the best of our knowledge, this study provides a comparative study between edible umbelliferous vegetable's volatile metabolites for quality evaluation and antimicrobial studies suggesting their uses as functional foods. However, some limitations can be taken into consideration for future work including the collection of the vegetables at different times to assess the seasonal variation in essential oils and the use of other extraction methods for the essential oils including headspace solid phase microextraction (HS-SPME).

## Material and methods

### Plant material

Four cultivated umbelliferous green vegetables viz*. Petroselinum crispum* L. (parsley)*, Anethum graveolens* L*.* (dill), *Apium graveolens* L. (celery), and *Coriandrum sativum* L. (coriander) were collected from local farms in Qualuob, El-Qualuobia governorate, Egypt, during May and June 2022. The plants were botanically collected identified by Prof. Dr. Rim Hamdy, Botany Department, Faculty of Science, Cairo University, Egypt. A voucher specimen has been deposited at Pharmacognosy Department, Faculty of Pharmacy, Egyptian Russian university No = 11CE6/22, 12PA6/22, 13CR6/22, and 14DL6/22. The methods in plant collection and experimentation were carried out in accordance with the IUCN Policy Statement on Research Involving Species at Risk of Extinction and the Convention on the Trade in Endangered Species of Wild Fauna and Flora.

### Extraction

The dried samples are coarsely grinded separately. The powdered plant (100 g each) was extracted with *n*-hexane (250 mL × 3) with cold sonication (using Biomall sonicator, India) for volatile extraction. The *n*-hexane is used as a solvent for its attributes such as simple recovery, and non-polar nature. The *n*-hexane extract was filtered and concentrated under reduced pressure using Rotary evaporator (Hahin-shin, Japan) at 40 ºC till concentration and complete evaporation of *n*-hexane solvent to yield concentrated extract with different weights for parsley (5 g), dill (5.5 g), celery (5.2 g), and coriander (4.9 g). The extracted oil of the tested plants was used for GC–MS analysis.

### GC–MS analysis

GC–MS analysis was performed at Pharmacognosy Department, Faculty of Pharmacy, Ain Shams University, Cairo, Egypt. Mass spectra were recorded using Shimadzu GCMS-QP2010 (Koyoto, Japan) equipped with Rtx-5MS fused bonded column (30 m × 0.25 mm i.d. × 0.25 μm film thickness) (Restek, USA) equipped with a split–splitless injector. The initial column temperature was kept at 50 °C for 3 min (isothermal) and programmed to 300 °C at a rate of 5 °C/min and kept constant at 300 °C for 10 min (isothermal). The injector temperature was 280 °C. Helium was used as a carrier gas with a flow rate of 1.37 mL/min. All the mass spectra were recorded applying the following condition: (equipment current) filament emission current, 60 mA; ionization voltage, 70 eV; ion source, 220 °C. Diluted samples (1% v/v) were injected with split mode (split ratio 1: 15).

### Metabolites identification and PCA data analyses

Identification of volatile chemical composition was performed by comparing their retention indices (RI) and retention times (RT) in relation to *n*-alkanes (C6-C20), mass matching to NIST 11.0, WILEY library database and with the available standards. Peaks were first deconvoluted using AMDIS software (www.amdis.net) before mass spectral matching^23,54^. Data were then subjected to principal component analysis (PCA), hierarchical clustering analysis (HCA) using SIMCA-P version 13.0 software package (Umetrics, Umeå, Sweden)^19^.

### Antibacterial activity

#### Tested microorganisms

The microorganisms were obtained from the Regional Center for Mycology and Biotechnology, Al-Azhar University, Egypt. Gram-positive bacteria *Staphylococcus aureus* ATCC 25923, *Bacillus subtilis* RCMB 015 (1) NRRL B-543, *Bacillus cereus* RCMB 027 (1), *Streptococcus mutants* RCMB 017 (1) ATCC 25175, *Enterococcus faecalis* ATCC 29212. Gram-negative bacteria *Enterobacter cloacae* RCMB 001(1) ATCC 23355, *Eschericha coli* ATCC 25922, and *Salmonella typhimurium* RCMB 006.

#### Agar diffusion method of well diffusion

The antibacterial activity was carried out according to previous reported studies with some modifications^44^. The culture medium is Mueller–Hinton agar recommended by National Committee for Clinical Laboratory Standards. The standard antimicrobial agent was gentamicin; as an antibacterial agent obtained from the Regional Center for Mycology and Biotechnology, Al-Azhar University, Egypt. The sterilized media was poured onto the sterilized petri dishes (20 mL each petri dish) and allowed to solidify. Wells of 6 mm diameter were made in the solidified media with the help of sterile borer. A sterile swab was used to evenly distribute microbial suspension over the surface of solidified media and solution of the tested samples were added to each well with the help of micropipette. The plates were incubated at 37 °C for 24 h. and The experiment was carried out in triplicate and zones of inhibition were measures in mm. scale.

#### Minimum inhibitory concentration (MIC)

The MIC was determined by the broth micro dilution method using 96-well micro-plates^44^. The inoculum of the microbial strains was prepared from 24 h. broth cultures and suspensions were adjusted to 0.5 McFarland standard turbidity. Each sample (1.0 mg) was dissolved in DMSO (1 mL) to obtain 1000 µg/mL stock solution. A number of wells were reserved on each plate for positive and negative controls. Sterile broth (100 µL) was added to the well from row B to H. the stock solutions of samples (100 µL) were added to the wells in row A and B. then, the mixture of samples and sterile broth (100 µL) in row B was transferred to each well in order to obtain a twofold serial dilution of the stock samples. The inoculums (100 µL) were added to each well and a final volume 200 µL was obtained in each well. Plates were incubated at 37 °C for 24 h. Microbial growth was indicated by the presence of turbidity of the well. The lowest concentration showing no growth was taken as the minimum inhibitory concentration (MIC).

### Statistical analysis

The results were displayed as average ± standard deviation of the mean (SD). The results of antibacterial activity were statistically analyzed using one-way analysis of variance (ANOVA), in which our findings are displayed as mean ± standard deviation (SD). Values with p < 0.05 are well-thought-out significantly different.

### Ethics approval and consent to participate

The plants were botanically identified and collected by Prof. Dr. Rim Hamdy, Professor of Plant Taxonomy, Botany Department, Faculty of Science, Cairo University, Egypt. A voucher specimen has been deposited at Pharmacognosy Department, Faculty of Pharmacy, Egyptian Russian university No = 11CE6/22, 12PA6/22, 13CO6/22, and 14DL6/22.

### Plant ethics

The methods in plant collection and experimentation were carried out in accordance with the IUCN Policy Statement on Research Involving Species at Risk of Extinction and the Convention on the Trade in Endangered Species of Wild Fauna and Flora.

The permissions of the plants collection were obtained in accordance with the guidelines prescribed by the American Society of Plant Taxonomists and adopted by the institutional research committee.

## Data Availability

All data generated or analyzed during this study are included in this published article.
