# Author Correction: Phytochemical investigation of the *n*-hexane-extracted oil from four umbelliferous vegetables using GC/MS analysis in the context of antibacterial activity

**DOI:** 10.1038/s41598-024-63713-5

**Published:** 2024-06-06

**Authors:** Mostafa H. Baky, Eman M. El‑Taher, Dina M. Y. El Naggar, Mostafa B. Abouelela

**Affiliations:** 1 Pharmacognosy Department, Faculty of Pharmacy, Egyptian Russian University, Badr City, 11829 Cairo Egypt; 2 Pharmacognosy Department, Faculty of Pharmacy for Girls, Al Azhar University, Cairo, Egypt

Correction to: *Scientific Reports* 10.1038/s41598-024-60631-4, published online 08 May 2024

The original version of this Article contained an error in Affiliation 1, which was incorrectly given as ‘Pharmacognosy Department, Faculty of Pharmacy for Girls, Egyptian Russian University, Badr City 11829, Cairo, Egypt’. The correct affiliation is listed below:

Pharmacognosy Department, Faculty of Pharmacy, Egyptian Russian University, Badr City 11829, Cairo, Egypt.

The original Article has been corrected.

